# Occupational health professionals’ and HR specialists’ perceptions of telemental health services in occupational health care settings: A qualitative study

**DOI:** 10.1177/20552076241297409

**Published:** 2025-01-06

**Authors:** Elina Kervinen, Lauri Vähätalo, Anna Siukola, Tiia Reho, Klas Winell, Riitta Sauni

**Affiliations:** 1 Faculty of Medicine and Health Technology, 7840 University of Tampere, Tampere, Finland; 2 Pihlajalinna, Tampere, Finland; 3 Conmedic Oy, Espoo, Finland

**Keywords:** Occupational health, mental health, telehealth, digital health, digital and remote health services

## Abstract

**Objective:**

The rise in mental health-related work disability pensions highlights the need for more research on how occupational health care (OHC) can support mental health, including the use of telehealth (TH) services in mental health care.

**Methods:**

The research, employing a descriptive qualitative approach through interviews (*n* = 42), focused on experiences of professionals from a private OHC service provider in Finland and human resource representatives (HRRs) of OHC client companies. Inductive content analysis was used to analyze the data.

**Results:**

Our research suggests that TH services provided by OHC can enhance access to care and expedite the initiation of work ability support, particularly in mental health cases. However, potential challenges include a perceived sense of distance, superficiality in interactions, and difficulty in forming a comprehensive understanding due to few non-verbal cues.

**Conclusion:**

A combined approach of TH and face-to-face services is suggested to provide flexible, and individualized support. Further studies on remote low-threshold discussion mental health services and studies comparing TH and face-to-face services are advised.

## Introduction

Mental health problems significantly impact the economy through increased absenteeism and reduced productivity.^1^ In Finland, over half of disability pensions and sickness absences in 2022 were related to mental health problems,^2,3^ with mental health costs accounting for 5.3% of gross domestic product (GDP) in 2018.^4^ The primary aim of occupational health care (OHC) is to support work ability and prevent disability, in collaboration with workplaces,^5^ as regulated by the Occupational Health Care Act (1383/2001).^6^ Under the act employers are required to offer preventive OHC to employees and may voluntarily provide curative care.^6^ In 2021, preventive OHC was offered to almost two million employees in Finland. In addition, curative care was available to 94% of those who received OHC services.^7^ This curative care may also include access to mental health services (for example psychologist) and telehealth (TH) services. The contents of employers’ voluntary curative contract may vary between workplaces and OHC service providers. Employers can obtain OHC services from municipalities, private service providers, or other service providers.^6,8^ In 2021, private service providers provided OHC services to 88% of all entitled to OHC services.^7^

The high prevalence of mental health problems has prompted the social and health care sector to explore new methods to enhance service availability and reduce costs. One proposed solution is increasing the use of telemental health (TMH) services,^9,10^ which previous studies have shown to be both accessible and cost-effective.^11,12^ In this study, TH services encompass all services—such as patient examination, diagnosis, monitoring, treatment, and decision-making—using information and communication technology. TMH services specifically refers to mental health services delivered via information and communication technology.^13^

Overall, TMH services are well received, and are considered comparable with face-to-face health services in many situations.^14–16^ There is also evidence that individual therapy, psychiatry visits, and group therapy sessions for people with major depression conducted via real-time audio and visual communication are as effective as face-to-face treatment.^17^ In addition to advantages there are also some disadvantages related to TMH services, such as, users having poor technological skills or unequitable access to TMH services^18,19^ and unsuitability to some situations for example due to poor communication.^20^ Philippe and colleagues caution against using TMH services for conditions like schizophrenia, bipolar disorder, and personality disorders.^21^ The COVID-19 pandemic spurred the development and use of TH services.^22,23^ In Finland, the OHC sector is argued to be at the forefront in the development of TH services, which is evidenced by the wider use of TH services in the OHC sector than other healthcare sectors.^24,25^ Kyytsönen and colleagues found that 32% of working-age individuals used some form of TH service provided by OHC during 2020–2021, compared to only 12% for public health care services.^24^ In 2022, about 50% of all OHC interactions occurred via remote consultations, phone calls, video conversations, chat, email, or other digital services.^25^

TMH services have been widely studied^26,27^ and there is some research on utilizing TH services in the OHC setting.^28,29^ The ever-growing amount of mental health-related work disability pensions calls for more research on OHC's possibilities to support mental health. This also requires more knowledge about the TMH services provided by the OHC.

### OHC collaboration

Collaboration between OHC service providers, employers, and employees is a crucial aspect of work ability support, and sickness absence and disability prevention.^30–32^ This involves developing support models for work ability, such as early intervention and return-to-work practices and monitoring their implementation.^33,34^ Effective collaboration can lead to earlier support for work ability issues, enhance the treatment of employees with mental health problems, and reduce related sickness absences and associated costs.^34,35^ At the workplace, top management decides on contracts and goals for OHC services, aligning them with strategic needs.^36^ A human resource manager/representative (HRR) usually coordinates OHC collaboration and liaises with OHC representatives and employees. Supervisors monitor and assess employees’ work ability and participate in workplace surveys and risk assessments. Employees provide feedback and needs for collaboration, with an employee representative disseminating information among workers.^36^ Both the entire workplace and individual employees of workplaces may be considered OHC clients. In this research an OHC client refers to individual employees.

OHC nurses facilitate the collaboration between OHC service providers and workplaces’ HRRs. OHC nurses also organize the internal activities of the multidisciplinary team (usually consisting of OHC nurse, OHC physician, OHC physiotherapist, OHC psychologist).^36^ An OHC physician is a medical expert within the multidisciplinary team who works closely with workplace representatives to promote employees’ health, safety, work ability, wellbeing, and assess risks. OHC physiotherapists and OHC psychologists participate together with other OHC professionals in workplace inspections, work ability assessments, rehabilitation, and support processes for work ability and their role is particularly emphasized in workplaces where either physiological or psychosocial factors significantly impact employees’ work ability and wellbeing.^36^

### Telehealth services provided by OHC service providers and external companies

OHC service providers offer both in-person and remote services, including guidance, counselling, short-term therapy, workplace investigations, work ability assessments, and negotiations via chat, phone, email, or video.^7,22^ They also provide digital tools such as appointment scheduling, well-being monitoring, online health questionnaires, electronic patient registries, and low-threshold mental health discussions (that in this study is managed by an independent unit).^37–40^ External companies similarly offer remote mental health services like counselling, career coaching, and support via phone, chat, and video.^41,42^

## Aim and research questions

Through qualitative interviews with OHC professionals and HRRs of OHC client companies, we aim to expand knowledge on the utilization and suitability of TMH services provided by OHC service providers in managing mental health problems among their clients in relation to work ability support.

Our research was guided by the following question:

- What are the positive experiences and challenges faced by HRRs of OHC client companies and OHC professionals regarding TMH services provided by OHC, in relation to work ability support?

## Methods

Our study evolved from ongoing research into how telehealth services within OHC settings can support and sustain work ability (a manuscript currently in progress). During our initial exploration, we discovered a narrative among interviewees regarding the potential of these services in addressing mental health challenges that threaten the work ability of OHC clients, focused separately on this area.

This research utilized a descriptive qualitative approach, through individual interviews with OHC professionals from a private service provider that offers OHC services in Finland and HRRs of OHC client companies; this type of research method is suitable when it is intended to understand and interpret people's experiences, views, motives, and meanings in a certain context.^43^ We chose to recruit participants from this specific OHC service provider because it is one of the largest and most widespread private service providers in Finland. This selection likely offers extensive knowledge and geographical variety for the study. To ensure the transparency of the research, the Consolidated criteria for reporting qualitative research (COREQ)-checklist was used in the reporting.^44^

## Participant recruitment

We recruited individuals with experience in TH services within a national OHC service provider. Convenience sampling and purposive sampling, which are ideal for identifying individuals with extensive knowledge on the topic with minimal effort,^43,45^were employed. Additionally, snowball sampling^46,47^ was utilized to complement the sample.

OHC service provider supervisors were contacted via email to disseminate information about the study and identify potential participants, who were then contacted by email or phone. Additionally, snowball sampling was employed, whereby interviewed professionals recommended additional colleagues. To capture the perspectives of OHC client companies, we recruited HRRs with extensive knowledge of TH services and their application in OHC client companies. OHC's client account managers assisted in identifying potential participants, who were contacted via email or phone. Snowball sampling was also used to recruit additional HRRs based on recommendations from interviewed OHC professionals. Based on Malterud and colleagues’ theory of information power,^48^ we estimated needing 20 OHC professionals and 20 HRRs, ultimately recruiting 28 professionals and 18 HRRs, which was deemed sufficient as saturation occurred.^49,50^ Four participants withdrew due to time constraints or perceived lack of expertise. Of the 42 recruited, 34 shared their experiences with TH services related to clients’ mental health. Participant details are provided in Table 1.

**Table 1.** Description of participants interviewed in the study, their self-explained information technology (IT) skills and stance to telehealth (TH).

Interview number	Duration of the interview (minutes)	Interviewee's industry sector	Interviewee's profession	Interviewees’ self-explained information technology (IT) skills and stance to telehealth (TH)
1	42	Social and health care services	HRR of OHC client companies	Good IT skills, positive attitude towards TH services, works in company where people have generally good IT skills and positive attitudes towards TH services
2	65	Industry	HRR of OHC client companies	Good IT skills, neutral attitude towards TH services, works in company where people have generally good IT skills and neutral attitude towards TH services
3	55	Public administration	HRR of OHC client companies	Good IT skills, positive attitude towards TH services, works in company where people have generally good IT skills and positive attitudes towards TH services
4	60	Social and health care services	HRR of OHC client companies	Good IT skills, neutral attitude towards TH services, works in company where people generally have varying IT skills and neutral attitude towards TH services
5	50	Service sector	HRR of OHC client companies	Good IT skills, positive attitude towards TH services, works in company where people have generally good IT skills and positive attitudes towards TH services
6	35	Service sector	HRR of OHC client companies	Good IT skills, neutral attitude towards TH services, works in company where people have generally poor IT skills and negative attitudes towards TH services
7	36	Technology services	HRR of OHC client companies	Good IT skills, positive attitude towards telehealth services, works in company where people have generally good IT skills and positive attitudes towards telehealth services
8	29	Industry	HRR of OHC client companies	Good IT skills, neutral attitude towards TH services, works in company where people have generally poor IT skills and neutral attitudes towards TH services
9	27	Service sector	HRR of OHC client companies	Good IT skills, positive attitude towards TH services, works in company where people have generally good IT skills and positive attitudes towards TH services
10	54	Industry	HRR of OHC client companies	Good IT skills, positive attitude towards TH services, works in company where people have generally good IT skills and positive attitudes towards TH services
11	32	Industry	HRR of OHC client companies	Good IT skills, neutral attitude towards TH services, works in company where people have generally good IT skills and neutral attitude towards TH services
12	45	Retail industry	HRR of OHC client companies	Good IT skills, neutral attitude towards TH services, works in company where people generally have varying IT skills and neutral attitude towards TH services
13	32	Retail industry	HRR of OHC client companies	Good IT skills, neutral attitude towards TH services, works in company where people have generally good IT skills and neutral attitudes towards TH services
14	60	OHC services	OHC nurse	Good IT skills, positive attitude towards TH services, offers both in-person receptions and remote receptions to clients
15	60	OHC services	OHC nurse	Okay IT skills, neutral attitude towards TH services, offers both in-person receptions and remote receptions to clients
16	59	OHC services	OHC nurse	Good IT skills, neutral attitude towards TH services, offers only remote receptions to clients
17	55	OHC services	OHC nurse	Good IT skills, positive attitude towards TH services, offers both in-person receptions and remote receptions to clients
18	46	OHC services	OHC psychologist	Good IT skills positive attitude towards TH services, offers both in-person receptions and remote receptions to clients
19	54	OHC services	OHC psychologist	Good IT skills, positive attitude towards TH services, offers both in-person receptions and remote receptions to clients four days per week and offers only remote receptions to clients one day per week
20	60	OHC services	OHC nurse	Okay IT skills, neutral attitude towards TH services, offers both in-person receptions and remote receptions to clients
21	50	OHC services	OHC psychologist	Good IT skills, positive attitude towards TH services, offers both in-person receptions and remote receptions to clients.
22	65	OHC services	OHC physician	Good IT skills, neutral attitude towards TH services, offers both in-person receptions and remote receptions to clients
23	37	OHC services	OHC physician	Good IT skills, positive attitude towards TH services, offers only remote receptions to clients
24	53	OHC services	OHC psychologist	Good IT skills, positive attitudes towards TH services, offers both in-person receptions and remote receptions to clients
25	32	OHC services	OHC physician	Good IT skills, neutral attitude towards TH services, offers both in-person receptions and remote receptions to clients
26	57	OHC services	OHC physician	Good IT skills, neutral attitudes towards TH services, offers mainly in-person receptions to clients
27	36	OHC services	OHC physician	Good IT skills, positive attitudes towards TH services, offers both in-person receptions and remote receptions to clients for days per week and offers only remote receptions to clients one day per week
28	75	OHC services	OHC physician	Good IT skills, neutral attitude towards TH services, offers both in-person receptions and remote receptions to clients
29	39	OHC services	OHC nurse	Okay IT skills, neutral attitude towards TH services, offers mainly in-person receptions to clients
30	31	OHC services	OHC physician	Good IT skills, positive attitudes towards TH services, offers both in-person receptions and remote receptions to clients
31	43	OHC services	OHC physician	Good IT skills, neutral attitudes towards TH services, offers both in-person receptions and remote receptions to clients
32	68	OHC services	OHC physician	Good IT skills, neutral attitudes towards TH services, offers both in-person receptions and remote receptions to clients
33	30	OHC services	OHC physician	Good IT skills, positive attitudes towards TH services, offers both in-person receptions and remote receptions to clients four days per week and offers only remote receptions to clients one day per week
34	30	OHC services	OHC psychologist	Good IT skills, positive attitude towards TH services, offers both in-person receptions and remote receptions to clients

*Note*. HRR = human resource representative; OHC = occupational health care.

## Data collection

Written informed consent was obtained from all participants before the study began. Signed consent forms were securely stored and will be destroyed after the study's completion. At the start of each interview, the interviewer(s) introduced themselves and explained the interview's purpose. None of the researchers had prior relationships with the participants. Interviews were conducted individually, with the first author conducting most interviews and the third author assisting in four. A semi-structured interview guide (Appendix 1 in the online supplemental materials, Interview guide), pilot-tested in three preliminary interviews, was used. The pilot interviews were not included in the data. Since our original purpose was not to study TMH services, but TH services in relation to work ability support (manuscript in progress), our interview questions did not include questions related to mental health. However, using TH services in mental health-related concerns came up during the interviews, and we focused on this area specifically. Interviews were conducted via video or telephone and recorded with consent. For three interviews where recording was declined, notes were taken and included in the data. Open discussion was encouraged, with follow-up questions used as needed and leading questions avoided.^51,52^ Transcriptions were verbatim, verified against the original recordings.

## Data analysis

Data were analyzed using inductive content analysis in a three-phase, iterative process.^51^ The first author conducted analysis. In the first phase of the analysis, the data were carefully read several times. In the next phase, the data that were significant in terms of the research question, in which the interviewees described their experiences of using TH services for mental health problems in relation to work ability, were carefully selected. These were named as meaning units. A condensed expression was attached to each meaning unit, which described the original meaning unit as accurately as possible, but in a condensed manner. A total of 151 condensed meaning units were formed. In the last phase, the condensed meaning units were classified into subcategories (*n* = 5) based on similarity and further into main categories (*n* = 2). All the categories derived from the data. Analysis relevance and consistency were ensured through team discussions, until consensus was reached, and results are presented with original citations for transparency and credibility.^53^

### Consideration of research ethics

The research received an ethical approval (Ethical statement 79/2023), and the study follows good scientific practice.^54^ Data were securely stored, accessible only to the research team members with regular security updates. Before publication, all identifiable information was removed, and findings were presented at an aggregated level. Pseudonymized data will either be transferred to the Finnish Social Science Data Archive (FSD) or securely destroyed upon the completion of data usage.

## Results

The analysis revealed two main categories: (1) experiences related to the use of TH services in OHC setting in matters related to mental health and (2) need for diverse services in mental health problems. The classification of these categories is detailed in Table 2.

**Table 2.** Classification of study results in the two main categories, their subcategories, and examples of condensed meaning units.

Condensed Meaning Unit (examples)	Subcategory	Main Category
TH services facilitate access to OHC especially when the client's concerns are related to mental health; there are more remote receptions available; TH services speed up the start of work ability support processes; discussions regarding work ability may feel safer for clients when they can do it at home, especially when the situation is related to mental health; expressing feelings may be easier at home than in face-to-face reception; some clients feel they can't talk in peace at home	TH services enable the work ability support to begin sooner, particularly for clients with mental health problems	Experiences in the use of TH services in OHC settings in matters related to mental health
TH services are not well-suited for all situations; TH services are not well-suited if the client is suspected of having a substance abuse problem; TH services are not suitable if the client is suicidal; superficial interaction in remote receptions hinders suitability for difficult mental health problems; experiencing distance in remote receptions hinders the suitability for difficult mental health problems; creation of the overall picture may be difficult in remote receptions; there are fewer non-verbal messages in remote receptions; video improves interaction	Superficial interaction in remote reception hinders the treatment of certain mental health problems	
Targeted investigations of psychosocial stressors in OHC client companies’ workplaces can be conducted remotely; if the problems hindering the client's work ability are related to mental health work ability assessments can be conducted in remote receptions; psychological tests cannot be done at a remote reception because the client's environment cannot be controlled well enough; short-term teletherapy is well received; for teletherapy to be successful, the client should be in a calm place; for the teletherapy to be successful, the client should have good technical equipment and connections; for the success of teletherapy, the client should have the camera on; face-to-face receptions are best for solving crises and conflict situations; interaction is better if you are present when solving crisis and conflict situations	Certain tasks of OHC professionals can be handled remotely	
A remote low-threshold discussion service that supports mental health eases the contact in OHC; a remote low-threshold discussion service that supports mental health speeds up access to treatment and the work ability support process; a remote low-threshold discussion service that supports mental health brings cost savings; a remote low-threshold discussion service that supports mental health reduces sickness absences; a remote low-threshold discussion service that supports mental health is separate from other OHC services; poor referral to the OHC treatment team from the remote low-threshold discussion service that supports mental health slows down the start of work ability support processes	Experiences related to the remote mental health discussion service provided by the OHC service provider	Need for diverse services in mental health problems
The remote discussion service that supports mental health, provided by a service provider outside the OHC, offers low-threshold help for challenges of everyday life; the remote low-threshold discussion service that supports mental health provided by a service provider outside of OHC enables faster access to treatment than similar services of OHC service providers; the remote low-threshold discussion service that supports mental health provided by a service provider outside of OHC does not involve heavy medicalization as similar services provided by providers of OHC services do; various low-threshold mental health supporting remote discussion services are necessary and their number and use will probably increase in the future; there is a need for various remote low-threshold discussion services that support mental health	Experiences related to external companies provided remote mental health discussion services	

### Experiences related to the use of Th services in OHC settings in matters related to mental health

#### TH services enable the work ability support to begin sooner, particularly for clients with mental health problems

Some HRRs of the OHC client companies and some OHC professionals felt that TH services can help people with mental health problems access OHC. Contacting via the electronic appointment system or chat may be easier for clients because they don't have to show their faces and there may be more anonymity, which may be particularly suitable for people with mental health problems. Remote receptions may often be more available (or sometimes the only available option when living far away from the service provider), which may also encourage clients to seek treatment:

…Telehealth services have increased seeking treatment, as they are available 24/7… (OHC physician, #33)

…Some people have a high threshold to call and make an appointment; chatting or making an appointment electronically is easier, especially when you don’t have to show your face and you can be more anonymous… (OHC nurse, #20)

…mental health issues, especially among young people, are increasing at a tremendous rate, and sick leaves related to them are also on the rise. In these situations, it is crucial that the individual receives help quickly. Digital services enable this. (HRRs of OHC client companies #7)

A few of the OHC physicians also described that in some situations, mental health challenges, such as anxiety and panic disorders, may be an obstacle for the use of face-to-face services, and in such cases TH services may be an opportunity to start the treatment:

…It's easier for anxious clients to communicate by phone, when they don't have to put on the video and don't have to come to the office. I remember a patient with panic disorder; we started treating him remotely, and after about a couple of weeks, he returned to work and was very happy to have received this remote assistance. (OHC physician, #33)

Some interviewees felt that TH services facilitate discussions about work ability, especially when clients’ problems are related to mental health. They noted that the distance inherent in TH services, combined with the comfort of one's home, can make it easier for clients to discuss distressing issues. Clients may also find it easier to express themselves at home:

…It might be easier for some, precisely because of the distance, to talk about something that is a little sensitive for them. (OHC psychologist #18)

…when there are psychosocial stress factors, and the patient is at home, it can be a safer place, and they may sometimes dare to cry more. Someone may have to harden themselves in a face-to-face reception, and they may feel comfortable crying only when they leave the room. (OHC physician, #28)

On the other hand, almost all OHC psychologists, also pointed out that for some people, their own home can be precisely such a place where they cannot talk openly:

…Some customers have said that: ‘I have children and a spouse here at home’. Or it may be difficult for a customer to find a place where they can talk in peace. Therefore, remote receptions may not be suitable in all cases. (OHC psychologist, #34)

Easy contact and access to treatment, as well as an easier discussion about work ability can speed up the start of the work ability support process, which may be important in terms of a faster return to work and thus societal productivity.

#### Superficial interaction in remote reception hinders the treatment in certain mental health problems

While remote receptions were generally viewed positively, some interviewees felt they were less suitable for cases where a client is suspected of having a substance abuse problem or suicidal thoughts. They noted that remote settings may obscure the client's genuine condition and restrict the depth of interaction necessary for an appropriate treatment:

…if someone is depressed or has suicidal thoughts, I try to get them a face-to-face reception with anyone as quickly as possible. (OHC nurse, #14)

…if it would be important to discuss feelings, remote receptions don't work so well. In remote receptions, we don't make direct eye contact but instead look into the camera. It's also easier for me to keep my distance from the emotional experience of the customers. The work may be less impactful due to this distance. (OHC psychologist, #18)

…people usually answer when asked, but sometimes the interaction remains a little more superficial if you would start working on those issues. (OHC psychologist #34)

However, in many cases, the use of a camera and a video connection were seen to improve interaction:

I prefer video, so you can see how the client is. On the phone, we only hear the voice, and sometimes you must interpret it a bit based on the voice, or ask: ‘What is happening there now? Did I interpret it correctly that you became sensitive?’ It's more challenging; video certainly makes it easier. (OHC psychologist #34)

Despite guidelines, some interviewees experienced that sometimes clients might not use camera, during remote receptions, for some reason, and OHC professionals may not encourage clients enough to use the camera:

A: Many keep the camera off during a remote reception, even if the goal is to see each other, in which case non-verbal communication would be better.

Q: If many people have their cameras off, do OHC professionals tell everyone to turn their cameras on?

A: It is true that it is generally not encouraged. A development idea could be to encourage it. This is perhaps more of a problem on the customer's side, that some don't have a camera or don't want to open the camera for one reason or another. (OHC psychologist, #34)

This underscores the need for OHC professionals to actively facilitate remote interactions, receive training on remote communication, and assess when face-to-face meetings are necessary.

#### Certain tasks of OHC professionals can be handled remotely

The interviewees discussed OHC activities that can and can't be done remotely. Several interviewees mentioned that targeted investigations of psychosocial stressors can be done remotely:

“A psychologist assesses the psychosocial stressors within the OHC client companies’ workplace and their impact on work ability. I start with an electronic survey on these stressors, followed by remote interviews where I concentrate on specific themes emerging from the survey findings. People have responded thoroughly to the electronic surveys; anonymity sometimes makes it easier for them to respond. (OHC psychologist, #18)

If we think about the assessment of psychosocial stress. It's different for a doctor and a nurse, but if a psychologist were to go to the workplace, say, to a factory, to assess psychosocial stress, it wouldn't work. (OHC psychologist #21)

In addition to conducting targeted investigations of psychosocial stressors remotely, many OHC physicians felt that if an employee has a mental health problem that affects employee's work, physicians can assess the employee's work ability by talking to them. In these cases, the work ability assessment can be conducted via a remote reception. Some OHC psychologists said they have done remote work ability assessments, but they felt that follow-up treatment is often better to do face-to-face. OHC psychologists also added that when they assess work ability, they need to do psychological tests. This is not practical remotely because they cannot control the clients’ environment, there might be disturbances, and clients might search answers from the internet:

Cognitive assessments made by a psychologist have their own difficulties, which is why they shouldn't be done remotely. You can't know if there's something in the client's environment that might affect the results. You don't know if the client has internet open and he or she could possibly look up what something means. (OHC psychologist, #18)

OHC psychologists generally supported short-term teletherapy but noted it is unsuitable for clients with severe depression or suicidal thoughts. They stressed the need to discuss the client's willingness to participate and ensure a calm environment and high-quality technical equipment for effective teletherapy:

…Clients have given good feedback on short-term teletherapy. Already during counselling visits, you can sense whether such short-term teletherapy would be suitable for the client. (OHC psychologist, #18)

Short-term therapy can be conducted through remote sessions. To ensure success, it's good to discuss with the client that they would seek out a quiet space where they can freely discuss matters without, for example, family members listening. Additionally, it's important for the client to have good technical equipment. (OHC psychologist, #19)

Some interviewees shared their views on resolving crises and conflicts in work communities via remote receptions. While some saw value in hybrid approaches, many generally felt that fully face-to-face meetings are more effective for managing crises and conflicts. According to them, physical presence enhances the handling of these situations.

### Need for diverse services in mental health problems

#### Experiences related to the remote mental health discussion service provided by OHC service providers

Many interviewees brought up the remote mental health discussion service offered by OHC providers. Most provided positive feedback, noting that it lowers the barrier to seeking help, speeds up access to support, and is cost-effective. Some HRRs from OHC client companies also observed a rise in mental health issues, underscoring the importance of such services. Additionally, one OHC nurse mentioned that this service could help individuals return to work earlier:

…we also have the option of short-term therapy through this digital service. We have received a lot of positive feedback about it. People have been helped to return to work faster, or sometimes sick leave hasn't even occurred, even though the threat has been there… (OHC nurse, #17)

However, because the remote discussion service operates as an independent unit within the OHC provider, it is not directly connected to other OHC services. As a result, professionals working in this service may not fully understand the impact of their client's mental health issues on work ability. This disconnect could lead to delays in referring clients to the OHC team, potentially postponing the start of necessary work ability support processes:

…Perhaps there is some mental health issue, and the connection to work and the work environment is not necessarily seen, which means that early support is not initiated. For example, a client has purchased these remote mental health discussion services provided by the OHC service provider. They are very separate from other OHC services. The client doesn't know that. (OHC psychologist #21)

#### Experiences related to remote mental health discussion services provided by external companies

External companies also offer remote low-threshold mental health discussion services, including counselling, career coaching, and low-threshold support for mental health challenges. Some HRRs of OHC client companies noted using these external services alongside OHC-provided ones, citing a perception of medicalization in OHC's remote low-threshold mental health discussion services:

…We also have an external company provided remote mental well-being support service. It's more of a counselling aid. If, for example, I were to go through a divorce, I can quickly get counselling with a psychologist there if I don't want sick leave and don't want to go through the process with OHC. In OHC, I see that there is the medicalisation that usually needs to be done before getting counselling. This is more of a low-threshold, quick support for everyday life challenges or similar situations. (HRR of OHC client company, #5)

The spontaneous mention of these external services may indicate a perceived need for them and a high level of usage. However, it also suggests that the services provided by OHC may not fully meet the needs of users as there is a clear preference for services from external providers, further emphasizing the need to develop OHC services.

## Discussion

The results indicated both positive and negative experiences related to the use of TH services in OHC settings in mental health-related situations and positive and negative experiences related to different remote low-threshold discussion services that support mental health and are provided by either OHC service providers or by external companies. We also compared the experiences between groups, but no significant differences were recognized.

According to our results, TH services were mostly viewed positively. Contacting OHC via electronic systems, chat, or applications can be particularly beneficial for individuals with mental health problems, as it may reduce stigma and social anxiety. Our results support previous research indicating that TH services may facilitate easier access to treatment.^55^ Specifically, those with anxiety or panic disorders may prefer TH services, aligning with previous study.^16^ Additionally, according to our results there may be more remote receptions available, further lowering the threshold for seeking help, evidenced also in previous studies.^11,12,56^

Many interviewees also believed that TH services could facilitate discussions about employees’ work ability, particularly when problems in employees’ work ability were related to mental health. Clients may feel safer discussing sensitive issues from home, where expressing emotions may be easier. Additionally, the sense of distance present in remote receptions might help clients to bring up sensitive topics, as has been shown earlier.^56,57^ LeBlanc and colleagues found that patients were more willing to share personal information because the distance and informality associated with TH services increased their comfort level.^56^ However, our findings also indicate that some individuals lack privacy or quiet spaces at home, which can hinder the discussion of sensitive matters. Prior studies have shown that background noise and the presence of others can inhibit open communication during remote receptions.^13,58,59^

In addition, many of the professionals interviewed found it challenging to understand clients’ overall situation during remote reception, due to few non-verbal messages. Interaction through screens was often perceived as superficial, diminishing the quality of discussions and creating a sense of distance that could hinder in-depth conversations. Gomez and colleagues noted that some patients perceive remote consultations as less personal than face-to-face meetings, which can discourage them from disclosing information.^57^ Consequently, interviewees felt that TH services might not be suitable for managing complex mental health problems, such as suspected substance abuse or suicidality. Kaihlanen and colleagues also highlighted that TH services are not ideal for addressing complex health problems due to the limitations in interaction.^60^ According to our findings, face-to-face receptions were deemed valuable for resolving internal workplace crises and conflicts and in situations related to complex mental health problems, such as suspected substance abuse or suicidal ideation. However, prior studies have demonstrated that TMH care is nearly as effective as face-to-face treatment for various mental health disorders, including severe ones.^17,61,62^ Differences between our findings and previous research may be attributed to variations in study designs, participant attitudes, and prior experiences. It is also advisable to offer face-to-face receptions alongside remote receptions, with OHC professionals determining when such consultations are necessary. This suggests that OHC professionals should receive training on how to assess the need for face-to-face receptions.

Our findings also suggest that using video during remote consultations can improve the quality of interaction. To ensure a successful remote experience and effective communication, professionals should encourage clients to use the camera during consultations. OHC professionals should also be trained in the specific aspects of remote communication. Pesonen and colleagues have also emphasized the active role of OHC professionals in fostering good communication during remote consultations.^63^

Positive experiences related to TH services, such as improved access to care, and facilitated discussions about work ability, may contribute to the earlier initiation of work ability support processes and thus earlier return to work.^33–35^ This can be crucial for productivity, as previous studies have shown.^33,34^ One potential way to improve access to care and promote earlier support for work ability is through low-threshold remote counselling services that provide mental health support and assistance with distressing issues. Many interviewees viewed these services positively and suggested that they could reduce costs by preventing sickness absences through faster access to care. However, the separation of these services from other OHC services might hinder the timely initiation of work ability support. Previous studies have highlighted the need for low-threshold remote mental health services.^22,35^ To our knowledge, this study is the first to describe experiences with such services, and we recommend further research on this topic.

### The reliability of the study

The sample for this study, recruited through supervisors and account managers of OHC service providers, may be less representative due to potential bias from personal relationships. However, the goal was to select individuals with deep subject knowledge, justifying the use of convenience, purposive, and snowball sampling. While these methods enriched the data, they may limit generalizability by focusing on a potentially narrow perspective.^43,45,59^ To enhance objectivity, the research team openly discussed preconceptions and used a semi-structured, pilot-tested interview guide with neutral, open-ended questions to ensure reliable data.^51,52^ The inductive content analysis followed a rigorous, well-documented process to improve reliability.^53,64^ However, findings should be cautiously applied beyond Finnish OHC services, as the data came from professionals and HRRs of a single provider, excluding individual client views. This is a common limitation in qualitative research.^51^

## Conclusion

OHC services play a crucial role in supporting the Finnish working-age population. Our findings suggest that OHC provided TH services can improve access to care and expedite work ability support, particularly for those with mental health problems. However, challenges such as perceived distance, superficial interactions, and limited non-verbal communication were noted.

An approach that combines digital, remote, and face-to-face services is recommended to ensure flexible and individualized care. For instance, incorporating remote low-threshold mental health discussion services can enhance accessibility. We recommend further comprehensive mixed-methods research on the impact of these services on work ability and productivity, as well as research that compares TH and face-to-face services.

## Supplemental Material

sj-docx-1-dhj-10.1177_20552076241297409 - Supplemental material for Occupational health professionals’ and HR specialists’ perceptions of telemental health services in occupational health care settings: A qualitative study Supplemental material, sj-docx-1-dhj-10.1177_20552076241297409 for Occupational health professionals’ and HR specialists’ perceptions of telemental health services in occupational health care settings: A qualitative study by Elina Kervinen, Lauri Vähätalo, Anna Siukola, Tiia Reho, Klas Winell and Riitta Sauni in DIGITAL HEALTH

sj-pdf-5-dhj-10.1177_20552076241297409 - Supplemental material for Occupational health professionals’ and HR specialists’ perceptions of telemental health services in occupational health care settings: A qualitative study Supplemental material, sj-pdf-5-dhj-10.1177_20552076241297409 for Occupational health professionals’ and HR specialists’ perceptions of telemental health services in occupational health care settings: A qualitative study by Elina Kervinen, Lauri Vähätalo, Anna Siukola, Tiia Reho, Klas Winell and Riitta Sauni in DIGITAL HEALTH
