# Supplementary material for: Occupational health professionals’ and HR specialists’ perceptions of telemental health services in occupational health care settings: A qualitative study
Source: Digit Health. 2025 Jan 6;11:20552076241297409. doi: 10.1177/20552076241297409 (PMC11705330; doi:10.1177/20552076241297409)
Supplement: sj-docx-1-dhj-10.1177_20552076241297409 - Supplemental material for Occupational health professionals’ and HR specialists’ perceptions of telemental health services in occupational health care settings: A qualitative study [file sj-docx-1-dhj-10.1177_20552076241297409.docx]

**S1 Appendix, Interview Guide**

**Questions for human resource representatives of occupational health care client companies**

First, may I ask if you have read the information letter and consent form related to the study? Would you like me to quickly go through their contents?

The aim of the study is to expand knowledge about the use of telehealth services provided by occupational health care (OHC) in supporting the work ability of their clients. By interviewing OHC professionals and human resource representatives (HRRs) of OHC client companies, we aim to bring out their views on the matter. Your participation in the study is voluntary and you can stop your participation at any time without giving a reason. All information you provide is confidential and you cannot be identified based on your answers. Next, can I ask for your consent to start the interview and record it?

1. Could you briefly describe your professional role?
2. How many years have you been in this role?

Theme 1 – Telehealth services and perceived benefits

1. What types of telehealth services provided by occupational health care service provider are available in your company?
2. Have you personally used these telehealth services provided by occupational health care, or have you heard about others' experiences with their use? How do you perceive these services?
3. What percentage of all occupational health visits do you estimate the use of telehealth services represents? What factors do you think contribute to this estimation?
4. How do you perceive yours, or your colleagues’ or occupational health care professionals’ capabilities to use telehealth services provided by occupational health care? User-related capabilities? Technical capabilities? Are they sufficient? If not, what additional resources would be needed to improve these capabilities?
5. How do you perceive these telehealth services to impact the health care costs in your company? Justify any potential cost savings or increases.
6. Do you consider these telehealth services to be value for money? Justify.
7. How do you perceive telehealth services provided by occupational health care to impact on employee’s presence at work or absenteeism? Have you noticed any changes in the number, duration, or frequency of employee’s sick leaves due to the use of telehealth services provided by occupational health? Is there evidence of time savings if employees don’t have to attend in-person appointments?
8. In what situations do you find telehealth services provided by occupational health care particularly beneficial or necessary? Justify.
9. In your opinion, in what situations do you find telehealth services provided by occupational health care unsuitable?

Theme 2 – Support for work ability in telehealth services

1. Do you have experience in using telehealth services provided by occupational health care in supporting work ability? How do you perceive them?
2. Is there some telehealth service or application specifically designed to support work ability? Have you used this? How do you perceive this?
3. Do you have experience with remote occupational health negotiations? How do you perceive these?
4. Do you have experience with low-threshold remote discussion service that supports mental health provided by occupational health care? How do you find it?
5. Does your company use any other low-threshold remote discussion services that supports mental health provided by third-party services providers? How do you find them? Why did you decide to use them?

Theme 3 – Other telehealth services

1. Do you have experience in updating an occupational health care plan remotely? How do you perceive it?
2. Do you have experience with electronic occupational health examinations or health surveys conducted remotely? How do you find them?
3. How do you deal with workplace assessments? Is there any part of it that is conducted remotely? How do you perceive these parts that are done remotely?
4. How do you perceive the referral to your own occupational health care team through telehealth services?
5. What kind of development suggestions do you have regarding telehealth services?
6. Is there anything else you would like to discuss? Is there anything we haven't addressed?

**Questions for occupational health care physicians**

1. Could you briefly describe your professional role?
2. How many years have you been in this role?

Theme 1 – Telehealth services and perceived benefits

1. What telehealth services do you provide to your clients? Do you know if your colleagues offer any other telehealth services to their clients?
2. How do you perceive these services, or have you heard how others perceive these services?
3. What percentage of all occupational health visits do you estimate the use of telehealth services represents? What factors do you think contribute to this estimation?
4. How do you perceive yours, or your colleagues’ or occupational health care customers’ capabilities to use telehealth services provided by occupational health care? User-related capabilities? Technical capabilities? Are they sufficient? If not, what additional resources would be needed to improve these capabilities?
5. In what situations do you find telehealth services provided by occupational health care particularly beneficial or necessary? Justify.
6. In your opinion, in what situations do you find telehealth services provided by occupational health care unsuitable? In what situations do you advise a patient to come for an in-person appointment?

Theme 2 – Work ability in telehealth services

1. How do you think remote contact affects initiating discussions about the need for work ability support? From both professionals and clients?
2. Do you have experience in using telehealth services provided by occupational health care in supporting work ability? How do you perceive them?
3. Do you feel you receive sufficient information about the patient's work-related issues in remote contact?
4. Would there be opportunities to utilize telehealth services more in supporting client’s work ability? Justify
5. What kind of work ability assessment do you conduct during remote appointments, if any? If not, why not? How do you perceive work ability assessments conducted remotely? How could this be improved?
6. Do you provide statements regarding client’s work ability remotely? If not, why? If yes, what kinds of statements regarding client’s work ability do you think you can provide remotely? How do you perceive this?
7. How do you perceive conducting B statements for disability benefits in remote appointments?
8. How do you perceive the following measures succeed remotely? (estimate on a scale of 1–5 where 5 is the best; if the estimate is 1 or 2, also the question, why?)

• Occupational health negotiation

• Work ability assessment

• Initiation visit for vocational rehabilitation

• Follow-up of partially work-capable individuals

• Assessment of rehabilitation needs

• Initiation visit for partial sickness allowance

• Initiation visit for medical rehabilitation

Theme 3 – Other telehealth services

1. Do you have experience in updating an occupational health care plan remotely? How do you perceive it?
2. Do you have experience in electronic occupational health examinations or health surveys conducted remotely? How do you perceive them?
3. How do you deal with workplace assessments? Is there any part of it that is conducted remotely? How do you perceive these parts that are done remotely?
4. How do you perceive the referral to own occupational health care team through telehealth services?
5. How do you perceive the increase of telehealth services affecting your overall working hours? What about your overall workload? And the content and meaningfulness of your work?
6. What kind of development suggestions do you have regarding telehealth services?
7. Is there anything else you would like to discuss? Is there anything we haven't addressed?

**Questions for other occupational health care professionals**

1. Could you briefly describe your professional role?
2. How many years have you been in this role?

Theme 1 – Telehealth services and perceived benefits

1. What telehealth services do you provide to your clients? Do you know if your colleagues offer any other telehealth services to their clients?
2. How do you perceive these services, or have you heard how others perceive these services?
3. What percentage of all occupational health visits do you estimate the use of telehealth services represents? What factors do you think contribute to this estimation?
4. How do you perceive yours, or your colleagues’ or occupational health care customers’ capabilities to use telehealth services provided by occupational health care? User-related capabilities? Technical capabilities? Are they sufficient? If not, what additional resources would be needed to improve these capabilities?
5. In what situations do you find telehealth services provided by occupational health care particularly beneficial or necessary? Justify.
6. In your opinion, in what situations do you find telehealth services provided by occupational health care unsuitable? In what situations do you advise a patient to come for an in-person appointment?

Theme 2 – Work ability in telehealth services

1. How do you think remote contact affects initiating discussions about the need for work ability support? From both professionals and clients?
2. Do you have experience in using telehealth services provided by occupational health care in supporting work ability? How do you perceive them?
3. Do you feel you receive sufficient information about the patient's work-related issues in remote contact?
4. Would there be opportunities to utilize telehealth services more in supporting client’s work ability? Justify
5. What kind of work ability assessment do you conduct during remote appointments, if any? If not, why not? How do you perceive work ability assessments conducted remotely? How could this be improved?

Theme 3 – Other telehealth services

1. Do you have experience in updating an occupational health care plan remotely? How do you perceive it?
2. Do you have experience in electronic occupational health examinations or health surveys conducted remotely? How do you perceive them?
3. How do you deal with workplace assessments? Is there any part of it that is conducted remotely? How do you perceive these parts that are done remotely?
4. How do you perceive the referral to own occupational health care team through telehealth services?
5. How do you perceive the increase of telehealth services affecting your overall working hours? What about your overall workload? And the content and meaningfulness of your work?
6. What kind of development suggestions do you have regarding telehealth services?
7. Is there anything else you would like to discuss? Is there anything we haven't addressed?

Thank you for your time!
